# Cincumol prevents malignant phenotype of colorectal cancer cell line HCT116 via inhibiting PI3K/AKT signaling *in vitro*

**DOI:** 10.1590/acb371201

**Published:** 2023-01-13

**Authors:** Gaowu Hu, Wenquan Chen, Wei Peng, Zhen Huang, Zhanlin Dong, Yongqing Cao

**Affiliations:** 1 MD. Shanghai University of Traditional Chinese Medicine – Department of Anorectal Medicine – Shanghai Traditional Chinese Medicine-Integrated Hospital – Shanghai, China.; 2 BD. Shanghai University of Traditional Chinese Medicine – Department of Anorectal Medicine – Longhua Hospital – Shanghai, China.

**Keywords:** Colorectal Neoplasms, Apoptosis, Phosphatidylinositol 3-Kinases

## Abstract

**Purpose:**

Colorectal cancer (CRC) is a common human cancer along with higher incidence and mortality, and this study aimed to identify the effect of cincumol on CRC and its potential mechanisms.

**Methods:**

CRC cell line HCT116 was used as the material. Cell proliferation was evaluated by CCK-8 assay, and cell migration was detected by scratch test and Transwell assay. TUNEL staining assay was used to evaluate cell apoptosis. The expression of target genes was detected by qualitative real-time polymerase chain reaction and western blot assays.

**Results:**

Cincumol significantly reduced the proliferative and migratory rate and enhanced apoptotic rate of HCT116 cells. Meanwhile, the elevated levels of RBUsuh, Nicd and Tace was also observed in cincumol-treated HCT116 cells. Moreover, our findings revealed that additional cincumol inhibited the expression of p-PI3K and p-AKT, suggesting the inhibition of PI3K/AKT signaling might be involved in the protective role of cincumol on the malignant phenotypes of CRC cells *in vitro*.

**Conclusions:**

Cincumol inhibited the malignant phenotypes of CRC cells *in vitro* through inactivating PI3K/AKT signaling, suggesting that cincumol might be a potential anti-CRC agent.

## Introduction

As one of the most common human malignant tumors, colorectal cancer (CRC) has a complicated pathogenesis^1^. According to the data from World Health Organization, the incidence and mortality rates of CRC in 2020 are 24.8 per 100,000 and 12 per 100,000^2^. Recently, surgery and chemotherapy are becoming one of the most efficient strategy for CRC treatment^3^. However, it is not satisfactory that the emergence of drug resistance has hindered the effectiveness of chemotherapy^4^. Hence, the identification of efficient anti-cancer active ingredients is necessary, which may help to develop new personalized treatment strategies.

Recently, more and more natural products are emerged as the main sources of anticancer drugs because of their better chemical diversity and relative low toxicity^5^^,^ 6. For example, acteoside was showed to inhibit tumor cells growth, alleviate inflammation, attenuate apoptosis in melanoma^7^. Salidroside inhibits cell proliferative rate and induces apoptosis of chronic myeloid leukemia cells^8^. There are many other natural products that have excellent anti-tumor activities, such as cucurbitacin E glucoside^9^, erianin^10^, and flavonolignan^11^. Cincumol is a guaiacene sesquiterpene and extracted from the root of *Rhizoma curcumae* 12.

Previous studies have reported that cincumol has multiple anti-tumor activities against different types of human cancers, and some of the molecular mechanisms also have been studied. For instance, cincumol inhibits the malignant phenotypes of pancreatic cancer cells *in vitro* and suppresses xenograft-tumor growth *in vivo* by regulating the miR-21-5p/SMAD7 axis^12^. Cincumol was reported to stimulate cell apoptosis and autophagy of human nasopharyngeal carcinoma cells, and then inhibited tumor development^13^. Cincumol was also demonstrated to inhibit the malignant phenotypes of glioma cells via attenuating long noncoding RNA FOXD2/As1 axis-stimulated EZH2 activation^14^. However, there were no relevant reports involved in the anti-tumor effect of cincumol in CRC, and we aimed to investigate its function and the potential molecular mechanisms.

In this study, we investigated the potential anti-CRC activity of cincumol, and we found that cincumol could significantly reduce cell viability and migration, while enhanced cell apoptosis of CRC cell line HCT116 *in vitro*. Meanwhile, additional cincumol led to an increase on the expression of RBUsuh, Nicd and Tace. Moreover, our findings suggested that the inhibition of PI3K/AKT signaling might account for the anti-CRC ability of cincumol *in vitro*, that was to say, cincumol was a potential anti-CRC drug that had the potential to be applied in clinical treatment.

## Methods

### Cell lines and treatment

Human CRC cell line HCT116 cells were purchased from Cell Resource Center of Shanghai (Shanghai, China), and grown within Dulbecco’s modified eagle medium (DMEM) containing 10% fetal calf serum (FBS, Gibco, United States of America) and 1% penicillin/streptomycin at 37 °C in 5% CO_2_ cell incubator. Cincumol (TMZ, HY-N0104) was dissolved in 1% dimethyl sulfoxide (DMSO), and those cells in control group (NC) was treated with 1% DMSO alone. For cincumol treatment, high concentration (20 μM) and low concentration (100 μM) was selected to treat HCT116 cells.

### CCK-8 assay

The viability was evaluated by using cell counting kit-8 detected cell viability (CCK-8, cat. No. HY-K0301, MedChemExpress). In brief, 2 × 10^5^ cells were seeded into 6-well plate and cultured overnight. After treating with 10 ug/mL cincumol for different times (0, 24, 48 and 72 h), cells were incubated with 10 μL of CCK-8 reagent for another 4 h. Finally, the absorption value at 450 nm was detected using a microplate reader. The experiment was replicated thrice.

### Scratch test

2 × 10^5^ cells were plated into 6-well plate and cultured overnight. The monolayer cells were scratched with 10-μL tip, and cells were washed with DMEM to remove cell debris. The fresh DMEM containing 10 μg/mL or 50 μg/mL cincumol was added into cells, and incubated for one, two and three days. Then, cells were observed by a microscope, and the scratch area was analyzed using The Cell Profiler software. The experiment was replicated thrice.

### Transwell assay

Cells were pre-treated with 10 μg/mL or 50 μg/mL cincumol for 24 h, and 2 × 10^5^ cells were plated into the upper chambers of the Transwell inserts uncoated with matrigel (Corning, United States of America). Then, the DMEM containing 20% FBS was added to the lower chambers. After 24 h of incubation, cells in lower chambers were washed with phosphate buffer saline, and then stained with 0.1% crystal violet. Finally, cells that have migrated into low chambers were photographed under an Olympus IX-71 microscope. The experiment was replicated thrice.

### Cell apoptosis assay

Cell apoptosis was evaluated by TUNEL staining assay with TUNEL Cell Apoptosis Detection kit (cat. no. FA201; TRANS). Briefly, 2 × 10^5^ cells were plated into 6-well plate and cultured overnight, then treated with 10 μg/mL or 50 μg/mL cincumol for 24 h. TUNEL staining was performed according to the instructions, and nuclei was stained by 5 μg/mL DAPI for 5 min at room temperature. The TUNEL-positive cells were counted and imaged using a fluorescence microscopy (magnification x100; Olympus Corporation). The experiment was replicated thrice.

### Quantitative real-time polymerase chain reaction

Total RNA was extracted using TRIzol reagent. The polymerase chain reactions (PCR) were performed with SYBR Premix Ex Taq II kit with GAPDH and U6 as the internal reference. The relative expression of target genes was calculated by 2^-DDCt^ method^15^. RBUsuh, F: 5’-CCAGCCTTACCTTTACCTACACAC-3’, R: 5’-AGGATACCACTGTGGCTGTAGATG-3’.Nicd, F: 5’-GTGCCGAACCAATACAACCCTC-3’, R: 5’-AGGCCCTGGTAGCTCATCATC-3’. Tace, F: 5’-AACTGGACCACCAGAGAATGGAC-3’, R: 5’-GAGATCCTCAAATGACTTGGCAGC-3’. GAPDH, F: 5’-AGGTCGGTGTGAACGGATTT-3’, R: 5’- TGTAGACCATGTAGTTGAGGTC-3’. The experiment was replicated thrice.

### Western blot

Total protein from cultured cells or tumors was extracted by RIPA lysis buffer. Approximately 30-μg samples were separated by 10% SDS-PAGE and transferred to polyvinylidene fluoride (PVDF) membranes. The membrane was incubated with primary antibodies including p-PI3K (1:500, ab182651, Abcam), PI3K (1:1,000, 4249, Cell Signaling Technology), AKT (1:1,000, 4691, Cell Signaling Technology), p-AKT (1:500, ab38449, Abcam), and GAPDH (1:2,000, ab9485, Abcam) overnight at 4 °C. Subsequently, the membranes were incubated with HRP-conjugated goat anti-rabbit secondary antibody (1:10,000; ab205718) for 1 h, and the bands of interesting targets were visualized with Bio-Rad imaging system. The experiment was replicated thrice.

### Statistical analysis

All data are presented as the means ± standard deviation (SD) method. Statistical analysis was performed using the Statistical Package for the Social Sciences (SPSS) software (version 18.0). Difference between two groups was tested by Student’s t-test, and difference between multiple groups was tested by one-way analysis of variance (ANOVA) followed by Tukey’s test to correct variance for multiple times on samples. For the data that do not conform to the normal distribution, the nonparametric Kruskal-Wallis’ test was applied. P < 0.05 was considered as the significant threshold.

## Results

### Cincumol significantly reduced HCT116 cell viability

**Figure 1:**
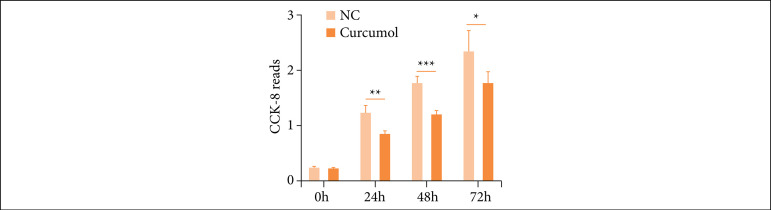
Cincumol reduced HCT116 cell viability. Ten μg/mL cincumol was added to treat HCT116 cells for different times, and the cell viability was detected by CCK-8 assay. Data are presented as means ± standard deviation,and the experiment was replicated thrice. One-way analysis of variance was used to evaluate thesignificance of each group, with Tukey’s test for corrected variance.

In order to explore the role of cincumol in CRC, we firstly detected the impact of it on HCT116 cell viability using a low dose of cincumol (10 μg/mL). As shown in Fig. 1, we found that 10 μg/mL cincumol significantly reduced the cell viability of HCT116 cells compared with NC group. The findings suggested that cincumol might have a potential anti-CRC ability.

NC: control group; *p < 0.05; **p < 0.01, ***p < 0.001.

### Cincumol significantly reduced HCT116 cell migration

**Figure 2:**
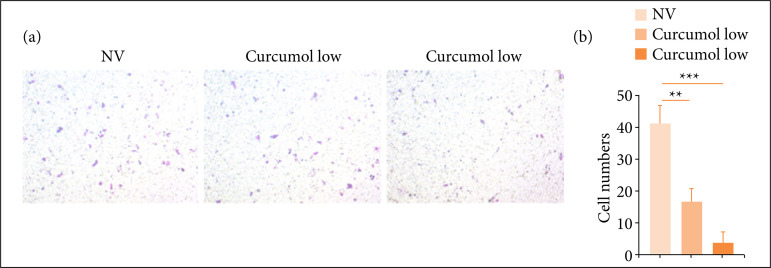
Cincumol inhibited HCT116 cell migration. **(a)** 10 μg/mL and 50 μg/mL cincumol was addedto treat HCT116 cells for 24 h, and the cell migrative numbers were evaluated by Transwell assay.**(b)** Quantitative analysis. Data are presented as means ± standard deviation, and theexperiment was replicated thrice. One-way analysis of variance was used to evaluatethe significance of each group, with Tukey’s test for corrected variance.

**Figure 3:**
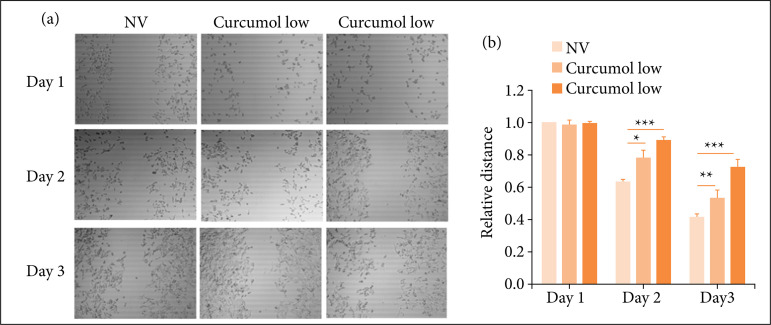
Cincumol inhibited HCT116 cell migration. **(a)** 10 μg/mL and 50 μg/mL cincumol was addedto treat HCT116 cells for one, two and three days, and the cell migrative capacity was detected byscratch test. **(b)** Quantitative analysis. Data are presented as means ± standard deviation,and the experiment was replicated thrice. One-way analysis of variance was used toevaluate the significance of each group, with Tukey’s test for corrected variance.

We investigated the impact of high and low dose of cincumol on HCT116 cell migration using two assays including Transwell assay and scratch test. As shown in Figs. 2a and 2b by Transwell assay, low and high dose of cincumol both reduced HCT116 cell migrative rate, and high dose of cincumol showed a more significant inhibitory effect. Similarly, by performing scratch test (Figs. 3a and 3b), we also found the inhibitory effect of two-dose cincumol on cell migration, as well as a stronger inhibitory capacity of high dose. These data further confirmed the anti-CRC ability of cincumol.

NC: control group; **p < 0.01; ***p < 0.001.

NC: control group; **p < 0.01; ***p < 0.001.

### Cincumol significantly enhanced HCT116 cell apoptosis

**Figure 4:**
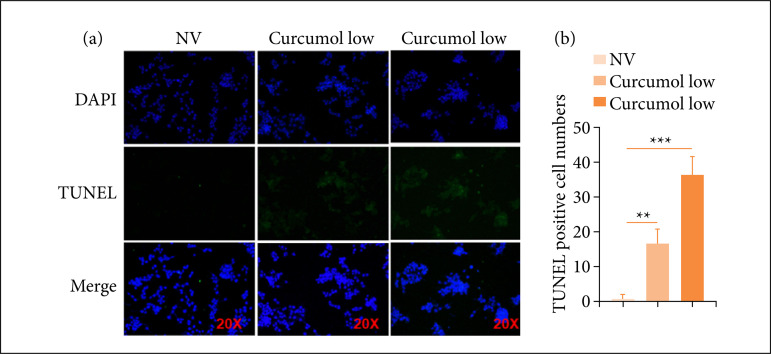
Cincumol enhanced HCT116 cell apoptosis. **(a)** 10 μg/mL and 50 μg/mL cincumolwas added to treat HCT116 cells for 24 h, and the cell apoptosis was measured by TUNELstaining assay. Magnification, 20×. **(b)** Quantitative analysis. Data are presented asmeans ± standard deviation, and the experiment was replicated thrice.One-way analysis of variance was used to evaluate the significanceof each group, with Tukey’s test for corrected variance.

**Figure 5:**
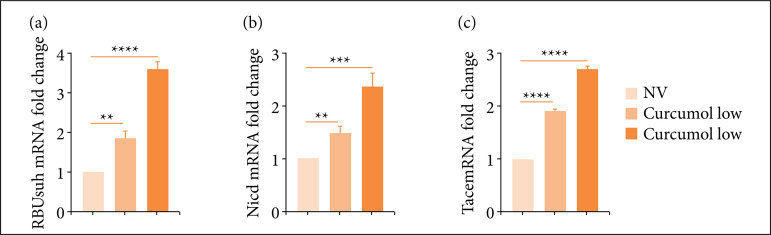
Cincumol reduced the expression of RBUsuh, Nicd and Tace in HCT116 cells. Ten μg/mL and 50 μg/mL cincumol was added to treat HCT116 cells for 24 h, and the expression change of**(a)** RBUsuh, **(b)** Nicd, and **(c)** Tace was detected by quantitative real-time polymerase chainreaction. Data are presented as means ± standard deviation, and the experiments werereplicated thrice. One-way analysis of variance was used to evaluate thesignificance of each group, with Tukey’s test for corrected variance.

We investigated whether cincumol played a role on cell apoptosis. As shown in Figs. 4a and 4b, two doses of cincumol both enhanced HCT116 cell apoptotic rate, and a high dose exhibited a strength effect in comparison to low dose. In addition, we also evaluated the expression of apoptosis-related genes in HCT116 cells after treating with cincumol, and we found that low dose of cincumol significantly elevated the expression of RBUsuh (Fig. 5a), Nicd (Fig. 5b) and Tace (Fig. 5c) in HCT116 cells, while high dose of cincumol further elevated the expression of these three genes (Figs. 5a-5c). These findings demonstrated that cincumol could significantly enhance HCT116 cell apoptosis to further prevent against CRC progression.

NC: control group; **p < 0.01; ***p < 0.001.

NC: control group; **p < 0.01; ***p < 0.001; ****p < 0.0001.

### Cincumol notably reduced the expression of STAT3/AKT signaling pathway

**Figure 6:**
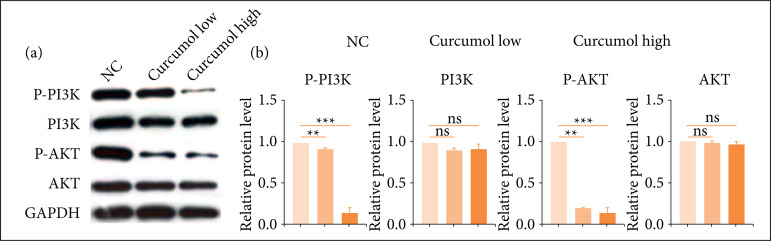
Cincumol inhibited the activation of PI3K/AKT signaling. **(a)** 10 μg/mL and 50 μg/mL cincumolwas added to treat HCT116 cells for 24 h, and the expression of p-PI3K, PI3K, p-AKT and AKT wasdetected by western blot. **(b)** Quantitative analysis of blots. Data are presented as means ± standarddeviation, and the experiment was replicated thrice. One-way analysis of variance was usedto evaluate the significance of each group, with Tukey’s test for corrected variance.

Finally, we investigated whether STAT3/AKT signaling was involved in the protective role of cincumol on CRC. By performing western blot (Figs. 6a and 6b), we found that both two doses of cincumol notably reduced the expression of p-PI3K and p-AKT, while exhibited no significant change on the expression of PI3K and AKT. All these results indicated that the inactivation of STAT3/AKT signaling might participate in the anti-CRC function of cincumol.

NC: control group; *p < 0.05; ***p < 0.001; **** p < 0.0001; ns: no clear difference.

## Discussion

Due to the higher incidence and mortality of CRC, investigators have paid more attention on its pathogenic mechanism, as well as the identification of novel anti-CRC drugs^16^^,^ 17. Previous studies revealed that many natural products can inhibit the development, the identification of more efficient anti-tumor drugs, and also the understanding of underlying molecular mechanisms is also necessary because of the complex pathogenic mechanism in CRC. In this study, we revealed for the first time that cincumol could significantly inhibit the proliferation and migration, while enhancing the cell apoptosis of CRC cell line HCT116 *in vitro*. Moreover, our results also suggested that cincumol might prevent malignant phenotypes of CRC cells *in vitro* through inhibiting the activation of PI3K/AKT signaling. Our findings indicated that cincumol might be a novel anti-cancer drug, which has the potential to be applied in clinic treatment.

Over the past decades, there were some natural products that were confirmed to have the anti-CRC effect under laboratory conditions. Meanwhile, most of these drugs act their anti-CRC activity through inhibiting cell proliferation, invasion, and migration, while promoting cell apoptosis. For example, bixin, an apocarotenoid from the seeds of *Bixa orellana*, was showed to attenuate the proliferation and motility of CRC cell lines (CaCO2 and SW480), followed by inhibiting CRC progression^18^. Granatin B and punicalagin, extracted from Chinese herbal medicine pomegranate peels, were also reported to induce cell apoptosis of CRC cells^19^. A recent study revealed that a large precious medicinal fungus, Sanghuang, also called *Sanghuangporus vaninii*, could lead to significant cell apoptosis of CRC cells through activating the intrinsic apoptotic pathway^20^.

Although the wide anti-tumor effect of cincumol in diverse human cancers have been reported, its potential role in CRC remains unclear. In the present study, we revealed that cincumol significantly inhibited the growth and migration of CRC HCT116 cells, and also stimulated cell apoptosis, then inhibited the progression of CRC *in vitro*. Our data suggested that cincumol might be a potential anti-CRC drug.

Further, our results indicated that PI3K/AKT signaling was closely involved in the anti-CRC activity of cincumol, and cincumol reduced the expression of p-AKT and p-PI3K, while exhibited no change on AKT and PI3K. In different types of signaling pathways, the phosphoinositide 3-kinase (PI3K)/AKT signaling plays a crucial role in human cancer, and aberrant activation may account for the serious tumorigenesis^21^^,^ 22.

At molecular level, overactivation of PI3K leads to elevated the level of PIP3, which in turn activates the downstream phosphorylation of AKT^23^. It has been reported that activation of AKT participated in the cancer development including inhibition of tumor cell apoptosis, promotion of invasion and migration in various cancers such as CRC^24^^,^ 25. These reports further confirmed that the activation of PI3K/AKT signaling might account for the anti-CRC effect of cincumol. In addition, except for PI3K/AKT signaling, there are some other important signaling pathways that was closely correlated with the progression of CRC, such as NF-κB-Nrf2 signaling^26^, Notch signaling^27^, MAPK signaling^28^, and JAK/STAT signaling^29^. We next plan to explore whether the anti-CRC activity of cincumol was related to the aberrant expression of these known signaling. In addition, the relevant suppressors of PI3K/AKT signaling pathway should be applied to demonstrate the participation of this signaling in the CRC progression.

## Conclusion

We revealed for the first time that cincumol could efficiently inhibit the development of CRC *in vitro* through attenuating the PI3K/AKT signaling pathway, followed by the inhibition of tumor cell growth and migration, as well as the promotion of cell apoptosis. Our findings suggested that cincumol was a novel anti-CRC drug, and potentially applied in clinical treatment for CRC.
